# High-Entropy Spinel Oxide Ferrites for Battery Applications

**DOI:** 10.1021/acs.chemmater.4c00085

**Published:** 2024-04-30

**Authors:** Ki-Hun Nam, Zhongling Wang, Jessica Luo, Cynthia Huang, Marie F. Millares, Alexis Pace, Lei Wang, Steven T. King, Lu Ma, Steven Ehrlich, Jianming Bai, Esther S. Takeuchi, Amy C. Marschilok, Shan Yan, Kenneth J. Takeuchi, Marca M. Doeff

**Affiliations:** † Energy Storage and Distributed Resources Division, Lawrence Berkeley National Laboratory, Berkeley, California 94720, United States; ‡ Institute of Energy: Sustainability, Environment and Equity, Stony Brook University, Stony Brook, New York 11794, United States; § Department of Materials Science and Chemical Engineering, Stony Brook University, Stonybrook, New York 11794, United States; ∥ Department of Chemistry, Stony Brook University, Stonybrook, New York 11794, United States; ⊥ Interdisciplinary Science Department, Brookhaven National Laboratory, Upton, New York 11973, United States; # National Synchrotron Light Source II (NSLS II), Brookhaven National Laboratory, Upton, New York 11973, United States

## Abstract

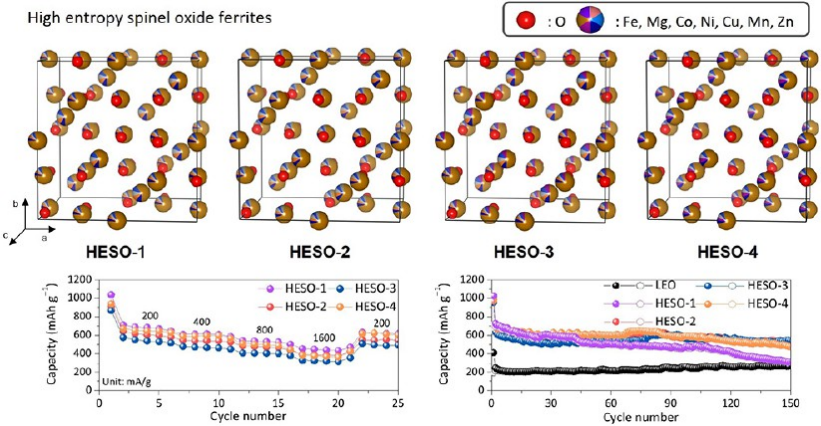

Four different high-entropy spinel oxide ferrite (HESO) electrode materials containing 5–6 distinct metals were synthesized by a simple, rapid combustion synthesis process and evaluated as conversion anode materials in lithium half-cells. All showed markedly superior electrochemical performance compared to conventional spinel ferrites such as Fe_3_O_4_ and MgFe_2_O_4_, having capacities that could be maintained above 600 mAh g^–1^ for 150 cycles, in most cases. X-ray absorption spectroscopy (XAS) results on pristine, discharged, and charged electrodes show that Fe, Co, Ni, and Cu are reduced to the elemental state during the first discharge (lithiation), while Mn is only slightly reduced. Upon recharge (delithiation), Fe is reoxidized to an average oxidation state of about 2.6+, while Co, Ni, and Cu are not reoxidized. The ability of Fe to be oxidized past 2+ accounts for the high capacities observed in these materials, while the presence of metallic elements after the initial lithiation provides an electronically conductive network that aids in charge transfer.

## Introduction

In recent decades, the widespread utilization of lithium-ion batteries (LIBs) in portable electronic devices and electric vehicles has catalyzed an intensive quest for alternative anode materials to supplant graphite. Despite graphite’s merits of high reversibility and cost-effectiveness, it is constrained by a limited capacity of 372 mAh g^–1^. Transition metal (TM) oxide-based conversion anodes, in contrast, offer a substantially higher theoretical capacity ranging from 600 to 1200 mAh g^–1^.^1^ These materials have undergone extensive investigation and are emerging as promising candidates for the next generation of LIBs. Unfortunately, they are plagued with issues such as poor electrical conductivity and considerable volume expansion during cycling, resulting in a marked degradation of capacity.

Recently, a new class of materials has been developed based on the concept of high-entropy, which relies on single-phase, multiple-element solid solutions.^2^ High-entropy materials (HEMs) are defined based on a configurational entropy value (Δ*S*) greater than 1.6R. Since the development of high-entropy alloys, various high-entropy oxides, nitrides, carbides, sulfides, and other compounds with lattice structures, such as rock-salt, spinel, perovskite, and fluorite, have been introduced and applied in various fields.^3^ Notably, they have exhibited unexpected and unusual properties compared to traditional materials. Among HEMs, high-entropy oxides (HEOs) have garnered significant attention due to their superior Li-ion storage properties based on the conversion reaction (MO + 2Li^+^ + 2e^–^ ⇌ M + Li_2_O). Sarkar et al. reported the synthesis of rock-salt structured HEO with the composition (Mg_0.2_Co_0.2_Ni_0.2_Cu_0.2_Zn_0.2_)O,^4^ which was utilized as a conversion-type LIB anode. During lithiation, cations of Co^2+^, Ni^2+^, Cu^2+^, and Zn^2+^ reduced their valence states, while Mg^2+^ maintained the rock-salt structure. However, spinel-structured HEOs are considered more promising LIB anodes due to their higher Li-storage properties compared to rock-salt HEOs. This is because the average oxidation state of metals in M_3_O_4_ is +2.67, while it is only +2 for MO. Thus, more electrons are transferred during the conversion reaction if metals are reduced to the elemental state.

Several spinel HEOs, including (FeNiCrMnMgAl)_3_O_4_^5^ and (Ni_0.2_Co_0.2_Mn_0.2_Fe_0.2_Ti_0.2_)_3_O_4_,^6^ have demonstrated high reversible capacities with stable capacity retention.

Despite these achievements in utilizing HEO anodes for LIBs, much effort is still needed to address fundamental research questions, including (1) whether the HEO is a single phase or not, (2) the site occupancies and distribution of multivalent transition metal cations, and (3) the Li-storage mechanisms involved in the conversion reaction during lithiation/delithiation. Nevertheless, there is limited research that provides clear answers to these fundamental questions.

Compared to the rock-salt structure, the spinel structure offers intriguing properties due to its large and complex unit cells, consisting of 32-anion sites surrounded by 24 cations organized in both octahedral and tetrahedral cages. Both ordered and disordered spinels have exhibited interesting electrochemistry in lithium-based systems.^7^ Ferrites, as representative spinel oxides, can be expressed as (M_1−γ_^2+^Fe_γ_^3+^)[Fe_2−γ_^3+^M_γ_^2+^]O_4_, where M can be Mg, Mn, Fe, Ni, Co, Zn, etc., and γ values range from 0 for normal or inversion spinels to 0 < γ < 1 for mixed spinels. Recently, Musicó et al. developed numerous different compositions of AB_2_O_4_ spinels (tetrahedral site, A = Mg, Mn, Fe, Co, Ni, Cu, and Zn; octahedral site, B = Cr or Fe) and X_3_O_4_ (X = Mg, Cr, Mn, Fe, Co, Ni, and Cu) and synthesized them through high-temperature sintering/calcination.^8^ Out of these compositions, only nine HEOs were successfully synthesized as single phases, and their structural and magnetic properties were investigated using X-ray absorption (XAS) and X-ray magnetic linear dichroism (XMLD). Antiferromagnetic ordering in spinels is temperature-dependent, and specific cations exhibit a preference for particular valence states. Therefore, the properties of high-entropy spinel oxides (HESO) depend on the combination of cations in spinels, with each cation favoring a 2+/3+ valence state preference. In terms of electrochemistry, the site occupancies and reduction of each cation during lithiation/delithiation should affect their electrochemical behavior. Thus, a structural understanding of HESOs could be the key to designing high-performance conversion-type LIB anodes.

Herein, we benchmarked HESO ferrites with four different compositions, each containing five or six metals. Materials for this study were designed to have disorder on the A site, based on preferential site occupancies for ions as detailed in the Musicó et al. paper, with the caveat that the complexity of compositions may result in site-mixing that could affect configurational entropy.^8^ Moreover, we used a different synthesis method, which could result in changed site occupancies. Short-range ordering can also reduce entropy and was not investigated here.^9^ While we do not have absolute proof that these materials are entropy stabilized, we are following contemporary nomenclature practice in the literature and expect that these materials are high-entropy oxides. These materials were synthesized via solution combustion synthesis (SCS) and evaluated as potential anode materials for LIBs. Notably, the HESO ferrites produced through the SCS process exhibited a high degree of crystallinity without requiring additional calcination. In addition, a two-component spinel (MgFe_2_O_4_) was fabricated using the same procedure to use as comparison. To gain a comprehensive understanding of their structure, we conducted in-depth structural characterization using synchrotron-based analyses, including X-ray diffraction (XRD) and XAS. Furthermore, we investigated the Li-storage mechanisms of HESO ferrites as conversion-type LIB anodes using XAS where the contributions of each redox active center were determined. The results revealed that HESO ferrite anodes displayed exceptional electrochemical performance, such as high reversible capacity, stable capacity retention, and rapid rate capability.

## Experimental Methods

### Chemicals

Metal nitrates, Mg(NO_3_)_2_·4H_2_O, Fe(NO_3_)_3_·9H_2_O, Co(NO_3_)_2_·6H_2_O, Ni(NO_3_)_2_·6H_2_O, Cu(NO_3_)_2_·3H_2_O, Zn(NO_3_)_2_·6H_2_O, Mn(NO_3_)_2_·4H_2_O precursors, and glycine were used to prepare ferrite HEO powders without further purification.

### Synthesis

The HESO ferrite (HESO-1, HESO-2, HESO-3, and HESO-4) powders were synthesized via the glycine-nitrate combustion method.^10^ To prepare the solution, equimolar amounts (0.02 mol) of five metal nitrates and Fe(NO_3_)_3_·9H_2_O (0.2 mol) were dissolved in deionized water with continuous magnetic stirring for 1 h. Subsequently, glycine was added to the solution (glycine/nitrate ratio = 0.56), and it was stirred for an additional 30 min. The homogeneous solution was then transferred to a stainless-steel beaker and placed on a hot plate. The solution was heated, gradually reaching a temperature of 300 °C, causing the water to evaporate, and forming a viscous gel. When the heating temperature reached 300 °C, the gel self-ignited within seconds, producing an ash-like combusted powder. This combusted powder was ground into fine particles using a mortar. For comparison, a low-entropy oxide (LEO, MgFe_2_O_4_) was synthesized in the same way.

### Characterization

Laboratory XRD data was collected on a Bruker D2 Phaser diffractometer with a Cu Kα source, equipped with a LynxEye detector. Synchrotron XRD of as-synthesized products was collected at the 28-ID-2 beamline at National Synchrotron Light Source II (NSLS-II). The detector was a 16-in. silicon panel equipped with a CsI scintillator. The X-ray wavelength was calibrated to 0.185736 Å. Rietveld refinements were performed with the GSAS-II software package.^11^ XAS of the pristine and *ex situ* HESO was conducted at NSLS-II using the 7-BM beamline at the Mn, Fe, Co, Ni, and Cu K-edges. Each XAS measurement represents a merge of multiple individual scans. The XAS spectra were aligned, merged, and normalized using Athena. The AUTOBK algorithm in Athena was used to reduce background contributions below *R*_bkg_ = 1.0 Å. The valence determination for Fe, Co, Ni and Cu was determined by linear combination fitting (LCF) analysis with the Athena software from the Demeter package.^12^ The selected energy range for LCF included −20 eV below to 30 eV above the edge energy for the least-squares fitting of normalized μ(E) spectra. The oxidation state of Mn was determined through the integral method as previously described, providing a more effective representation for Mn,^13^ where the value of edge energy represents the mean value of the energy in the edge region. The same method was applied to the spectra of standard materials (MnO, Mn_3_O_4_, Mn_2_O_3_, and MnO_2_) to establish calibration curves between edge energy and oxidation state of Mn and then used to determine the oxidation state of the Mn in the HESO samples (the details are described in the Supporting Information, see Figures S1–S3). Extended X-ray absorption fine structure (EXAFS) spectra fitting was carried out using Artemis, and structural models were calculated with FEFF6. The structural model was developed using a Fe_3_O_4_ structure with a *Fd*3̅*m* space group and was kept constant throughout the fitting of each K-edge. Each fit was conducted in a *k*-range of 2–10 Å^–1^ with a Hanning window (d*k* = 2) in *k*, *k*^2^, and *k*^3^*k*-weights simultaneously. An *R*-range of 1.0–3.7 Å was used in all samples. Additionally, *S*_0_^2^ parameter was determined from fitting the Fe_3_O_4_ standard,^14^ and this term was applied to all fits to account for the ratios of Fe atoms located at tetrahedral sites and octahedral sites in the pristine HESO samples.^15^

The elemental composition for each sample was determined using inductively coupled plasma-optical emission spectroscopy (ICP-OES) using a ThermoScientific iCap 6300. Scanning electron microscopy (SEM) characterization was carried out at 3 kV, and energy-dispersive X-ray spectroscopy (EDS) mapping images were collected at 20 kV using a ZEISS Crossbeam-340 instrument. Transmission electron microscopy (TEM) data including images and diffraction were acquired using a JEOL 1400 operated at 80 kV.

### Electrochemical Testing

The HESO powders were used as the active materials for the cathodes in coin cells utilizing lithium metal as the anode. The cathode was 70% HESO, 20% carbon black, and 10% sodium carboxymethyl cellulose (Na-CMC) dissolved in deionized water by mass. The average loading level of active material was maintained at 0.9–1.1 mg cm^–2^. An electrolyte of 1 M LiPF_6_ in 1:1 (v/v) ethylene carbonate (EC)/diethyl carbonate (DEC) electrolyte was used. Galvanostatic cycling was conducted in the voltage range of 0.3–3.0 V vs Li/Li^+^ at 30 °C at 200 mA g^–1^ using a Bio-Logic VMP3 potentiostat/galvanostat. Rate capability testing was also carried out using a MACCOR multichannel testing system at 30 °C. Rate capability was tested at several current densities, specifically, at 200, 400, 800, 1600, and then 200 mA g^–1^ successively for five cycles at each rate. Electrodes in the discharged and charged conditions for each sample were extracted from coin cells and retained under an inert atmosphere for XAS measurements. The XAS spectra were analyzed as described above.

## Results and Discussion

### Synthesis and Characterization

**Scheme 1:**
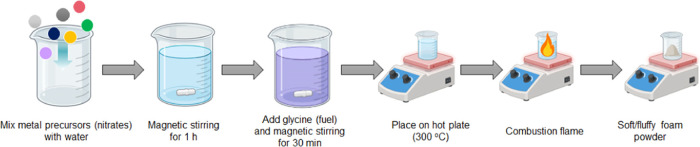
Solution Combustion Synthesis (SCS) Method for HESO Ferrite Powders

The HESO ferrite (HESO-1, HESO-2, HESO-3, and HESO-4) powders were synthesized via a SCS method, as shown in Scheme 1. This is a quick (about 2 h from start to finish) and moderately large scale (>10 g per batch) method compared with other synthesis techniques. MgFe_2_O_4_ (LEO) was also synthesized by the same method. All attempts to produce Fe_3_O_4_ by SCS failed, yielding Fe_2_O_3_ instead. The nominal compositions of the HESO powders are given in Table 1. The elemental composition was confirmed by inductively coupled plasma optical emission spectroscopy (ICP-OES) and was close to the nominal values.

**Table 1:** ICP-OES Results on HESO Ferrite Materials

sample	targeted composition	ICP-OES results
HESO-1	[Mg_0.2_Co_0.2_Ni_0.2_Cu_0.2_Fe_0.2_]Fe_2_O_4_	[Mg_0.21_Co_0.19_Ni_0.20_Cu_0.21_Fe_0.20_]Fe_2_O*_y_*
HESO-2	[Mg_0.2_Co_0.2_Ni_0.2_Cu_0.2_Zn_0.2_]Fe_2_O_4_	[Mg_0.20_Co_0.19_Ni_0.20_Cu_0.21_Zn_0.15_]Fe_2_O*_y_*
HESO-3	[Mg_0.2_Co_0.2_Ni_0.2_Cu_0.2_Mn_0.2_]Fe_2_O_4_	[Mg_0.21_Co_0.19_Ni_0.20_Cu_0.21_Mn_0.20_]Fe_2_O*_y_*
HESO-4	[Mn_0.2_Co_0.2_Ni_0.2_Cu_0.2_Fe_0.2_]Fe_2_O_4_	[Mn_0.20_Co_0.19_Ni_0.20_Cu_0.21_Fe_0.20_]Fe_2_O*_y_*

**Figure 1:**
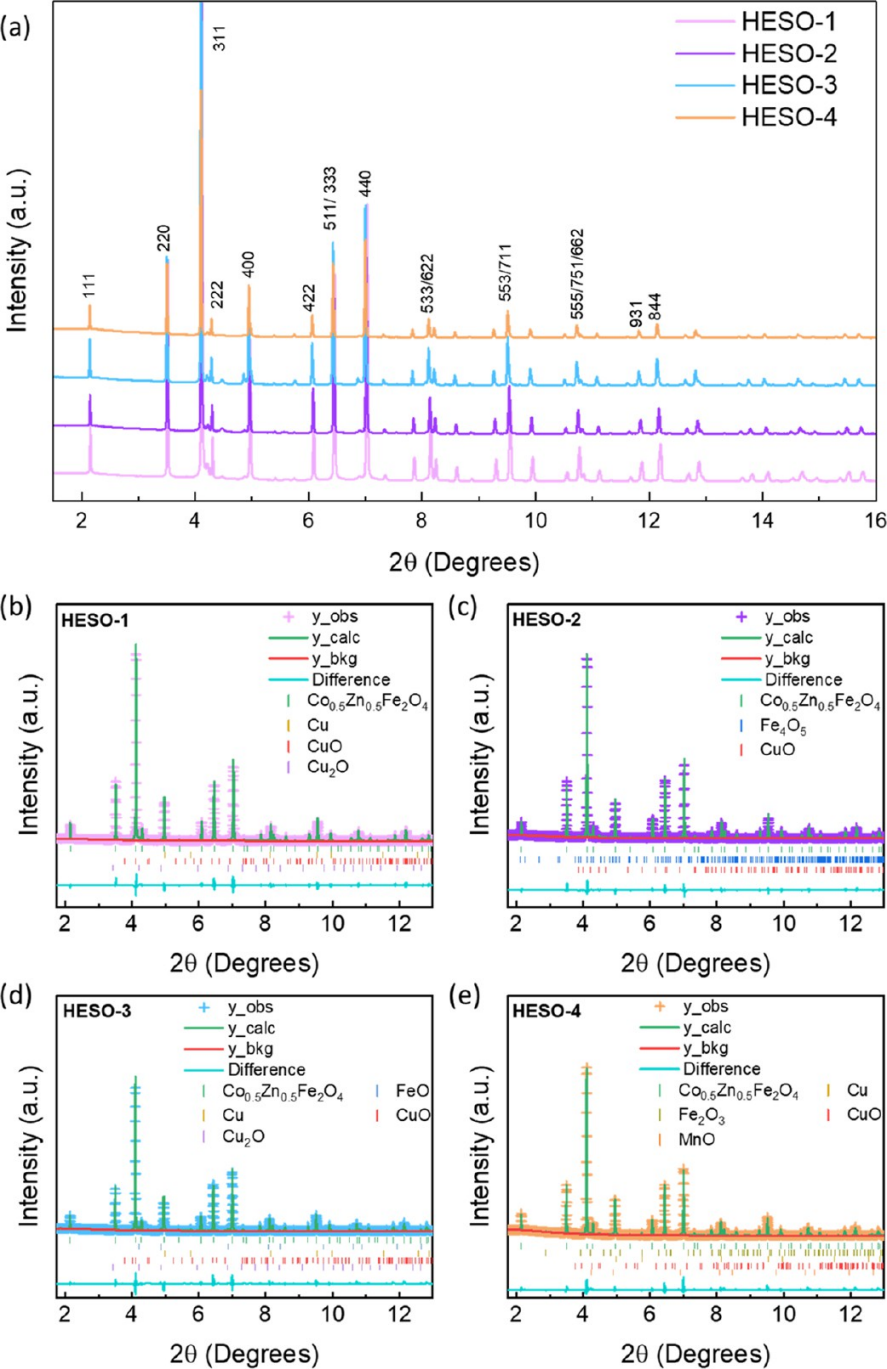
(a) Synchroton XRD patterns of HESO-1 (light purple), HESO-2 (purple), HESO-3 (light blue), and (d) HESO-4 (light orange). (b–e) Refinement plots of (b) HESO-1, (c) HESO-2, (d) HESO-3, and (e) HESO-4.

Synchrotron XRD patterns of the HESO compounds are presented in Figure 1, and a laboratory XRD pattern of MgFe_2_O_4_ is shown in Figure S4. All HESOs appeared to be highly crystalline, consisted primarily of cubic inverse spinel structures belonging to the *Fd*3̅*m* space group, and were indexed to Co_0.5_Zn_0.5_Fe_2_O_4_ (ICSD code: 184064). The plots are presented in Figure 1, and the refinement parameters of the main phases are presented in Table 2. The *a* lattice parameter increased from HESO-1 to HESO-2, as expected due to the replacement of Fe with the larger Zn cation, and increased further with Mn substitution in HESO-3, suggesting a fairly low oxidation state for this cation. In HESO-4, the *a* lattice parameter decreased slightly due to the substitution of Mg with the Fe cation. All of the HESO powders contained >92% of the spinel phase, with only small amounts of impurities (Table 3). HESO-1 contained the lowest amount of impurities (2.8%), while HESO-2 contained the highest (8%). For HESO-1, -2, and -3, the impurity peaks were indexed to different Cu and Fe oxides that may have formed during synthesis. HESO-4 contained an additional impurity phase that was indexed to MnO (3.9%). Additionally, the small peak at ∼5.75° was indexed to elemental Cu.

**Table 2:** Refinement Parameters from Synchrotron XRD Data for HESO Materials, Space Group *Fd*3̅*m*^a^

sample	nominal composition	*a* (Å)	*R*_wp_	GOF
HESO-1	[Mg_0.2_Co_0.2_Ni_0.2_Cu_0.2_Fe_0.2_]Fe_2_O_4_	8.3682(8)	8.87	0.06
HESO-2	[Mg_0.2_Co_0.2_Ni_0.2_Cu_0.2_Zn_0.2_]Fe_2_O_4_	8.3839(6)	7.97	0.03
HESO-3	[Mg_0.2_Co_0.2_Ni_0.2_Cu_0.2_Mn_0.2_]Fe_2_O_4_	8.4037(2)	10.79	0.06
HESO-4	[Mn_0.2_Co_0.2_Ni_0.2_Cu_0.2_Fe_0.2_]Fe_2_O_4_	8.4032(3)	8.30	0.02

a GOF is reduced Chi-squared.

**Table 3:** Phase Fractions of HESO Powders Determined from Rietveld Refinement of Synchrotron XRD Patterns

sample	spinel (%)	Fe_4_O_5_ (%)	Fe_2_O_3_ (%)	FeO (%)	Cu (%)	CuO (%)	Cu_2_O (%)	MnO (%)
HESO-1	97.2				0.3	1.0	1.5	
HESO-2	92.0	4.0				4.0		
HESO-3	94.2			1.5	1.7	1.2	1.4	
HESO-4	92.1		0.2		1.9	1.9		3.9

**Figure 2:**
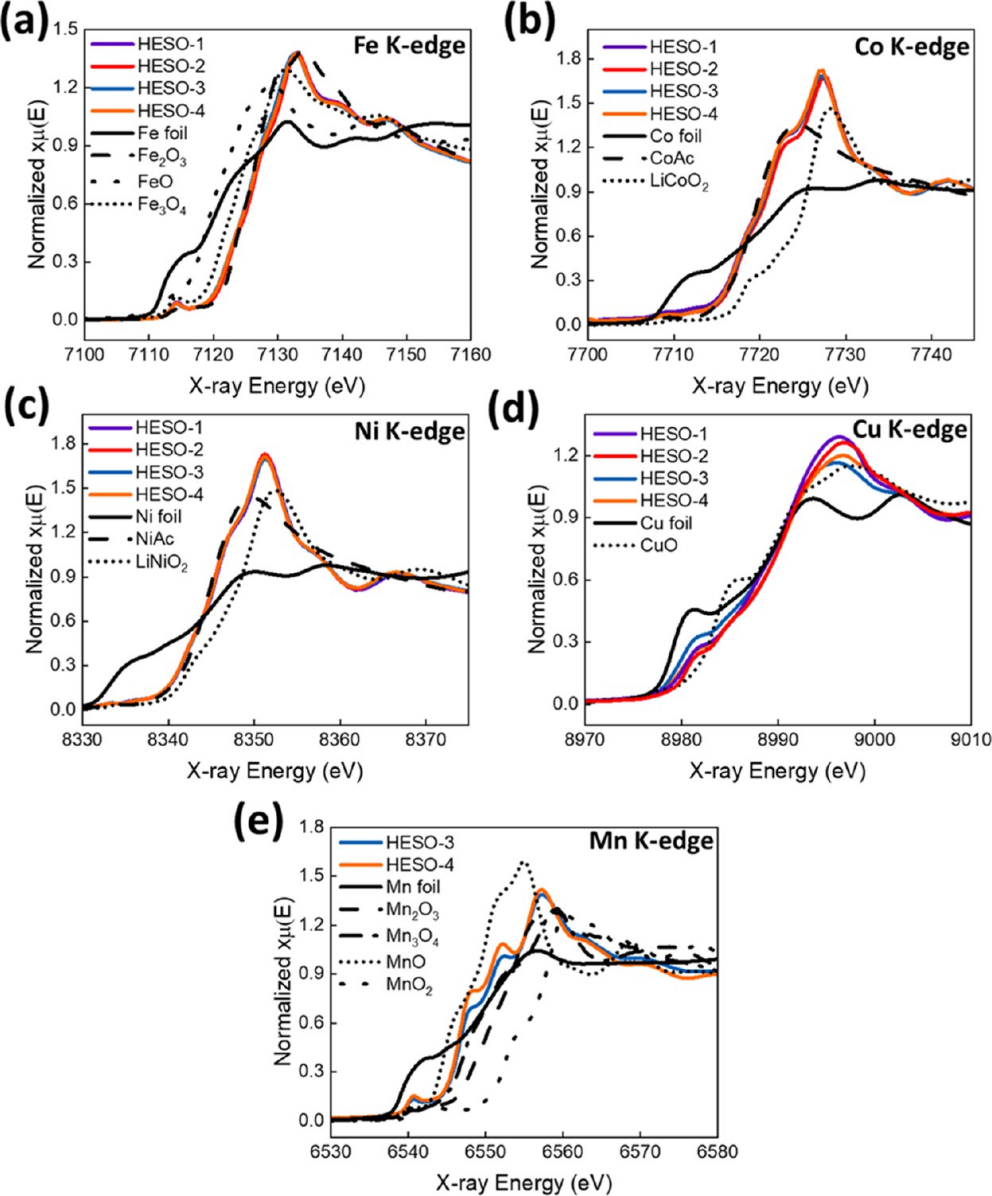
XANES spectra of HESO samples and standards at (a) Fe K-edge, (b) Co K-edge, (c) Ni K-edge, (d) Cu K-edge, and (e) Mn K-edge.

Fe, Co, Ni, Cu, and Mn K-edge X-ray absorption near-edge structure (XANES) spectra for the as-made HESO products were collected to determine the oxidation state of the metal centers (Figure 2). In the Fe-edge, Co-edge, and Ni-edge data (a, b, and c, respectively), all 4 samples exhibit approximately the same absorbance spectra; the absorption edges of all 4 samples appear at the same position, and the spectra differ only very slightly in absorption intensity. The edge positions in the Fe series correspond primarily to Fe(III), evidenced by their alignment with the Fe_2_O_3_ standard. The Co series exhibits 3 clear edges near 7717, 7722, and 7727 eV. The edges at 7717 and 7727 eV appear in similar positions as the Co acetate Co(II) and LiCoO_2_ Co(III) standards. However, the primary absorption edge at 7722 eV is not well-represented by the standards used for fitting, although the LiCoO_2_ standard does appear to also contain an absorption edge near this same position. The Ni series aligns well with the Ni acetate standard edge at 8343 eV, indicating an oxidation state near Ni(II). There is an additional edge located at 8348 eV, which is not present in the Ni acetate standard but is represented by the LiNiO_2_ Ni (III) standard, indicating the presence of some amount of Ni at an oxidation state greater than 2.0.

The Cu XANES measurements do not vary significantly in edge position but do differ significantly in absorption intensity in several locations across the XANES region. All samples exhibit an edge at 8981 eV, indicative of Cu(0). The intensity of the absorption at energies just above the absorption edge differs significantly, with samples HESO-3 and HESO-4 exhibiting greater absorption than samples HESO-1 and HESO-2. All samples then possess a secondary edge near 8992 eV, corresponding to Cu(II). The absorption intensity of each sample just above this edge again differs significantly, this time with HESO-1 and HESO-2 demonstrating greater absorption than HESO-3 and HESO-4. This suggests a greater component of Cu metal in the HESO-3 and HESO-4 samples than in the HESO-1 and HESO-2 samples, as also observed in the XRD refinements. The Mn XANES for the HESO-3 and HESO-4 exhibit at least 4 distinct absorption edges, suggesting a complex mixture of Mn oxidation states. The edges appear at 6540, 6547, 6552, and 6557 eV and correspond to Mn(0), Mn(II), Mn(III), and Mn(IV), respectively, as evidenced by comparison to the Mn metal, MnO, and Mn_3_O_4_ reference standards. Linear combination fitting was used to determine the oxidation states of the Fe, Co, Ni, and Cu metals at each state. The determination of the oxidation states of Mn has been reported as requiring additional consideration as the main absorption edge arises from the electric dipole-allowed transition from the 1s to 4p level.^16^ Here, three methods were compared for the determination of the Mn oxidation state: (1) determining the edge energy at the maximum point of the first peak of the first derivative of each spectrum, (2) determining the edge energy at the half-height of each spectrum, and (3) using an integral area to obtain the mean value of the edge energy in the edge region. In each method, spectra of MnO, Mn_2_O_3_, MnO_2_, and Mn_3_O_4_ standards were used to establish calibration curves between edge energy and the oxidation state of Mn, as discussed in the Supporting Information and shown in Figures S1–S3. The integral method yielded the highest linear correlation of the Mn standards and was used for the determination of the Mn oxidation states of the samples. Average oxidation states for the different metals in the HESO samples and their oxygen contents calculated using charge balance assumptions are summarized in Table 4.

**Table 4:** HESO Average Metal Oxidation States and Formulas from XANES Data

	oxidation states					
sample	Fe	Co	Ni	Cu	Mn	composition
HESO-1	2.91	2.08	2.20	1.83		[Mg_0.21_Co_0.19_Ni_0.20_Cu_0.21_Fe_0.20_] Fe_2_O_4.03_
HESO-2	2.92	2.19	2.15	1.98		[Mg_0.20_Co_0.19_Ni_0.20_Cu_0.21_Zn_0.15_]Fe_2_O_3.90_
HESO-3	2.89	2.08	2.15	1.64	2.43	[Mg_0.21_Co_0.19_Ni_0.20_Cu_0.21_Mn_0.20_]Fe_2_O_3.93_
HESO-4	2.87	2.10	2.17	1.29	2.24	[Mn_0.20_Co_0.19_Ni_0.20_Cu_0.21_Fe_0.20_]Fe_2_O_3.95_

Oxidation states for Mn and Fe for the two analogs of HESO-3 and HESO-4, measured by XAS, have also been reported in ref (8) as a function of temperature. The room temperature values are somewhat different from those reported here. For the material compositionally similar to HESO-3 at 300 K, Musicó et al. found an oxidation state of 2.43+ for Fe and 2.42+ for Mn. While the oxidation state of Mn in that study closely matches that for HESO-3 in this one, Fe is more oxidized in our material. The opposite is true for HESO-4; the Fe oxidation state was found to be 2.86+ in the earlier study, similar to our results, and that of Mn was 2.89+, higher than found here. This may be a consequence of the different synthesis methods used to make the samples. Combustion synthesis is so rapid that the products may not always be the thermodynamically favored phases, in contrast to solid-state synthesis, which proceeds close to equilibrium. Another factor may be the presence of minor impurity phases in HESO-3 and HESO-4 reported here, which result in the compositions of the main phases being slightly different from those of the ones reported earlier.

The extended X-ray absorption fine structure (EXAFS) region of XAS provides information about bond lengths and coordination of the ion being probed. To offer quantitative elucidation regarding the pristine structure of HESO samples, the EXAFS fitting was applied through the Artemis software. This facilitated the determination of the interatomic distances between the core metal Fe and its neighboring O or Fe atoms, as well as the percentage of tetrahedrally and octahedrally coordinated Fe atoms, as depicted in Figure S5. Detailed fitting parameters and results are comprehensively presented in Table S1. Best fits were obtained when half of the Fe in the HESO materials were located in tetrahedral sites and half in octahedral sites. While HESO-1 and HESO-4 were designed to have Fe on both sites, HESO-2 and HESO-3 were not. Thus, occupancies are somewhat different from the target expressed in the Introduction section.

**Figure 3:**
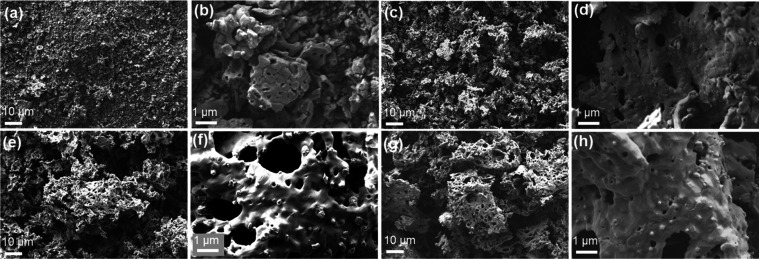
SEM images of (a, b) HESO-1, (c, d) HESO-2, (e, f) HESO-3, and (g, h) HESO-4.

The morphologies of HESOs were characterized by SEM and EDS, as shown in Figure 3. The HESO materials are composed of micrometer-sized particles with large pores or tunnels consisting of nanoparticles fused together. This is caused by the escape of gases during the rapid combustion process. Both the glycine:nitrate ratio (held constant for these syntheses) and the identities of the metals in the starting materials determine the temperature at which combustion occurs. The reaction temperature, therefore, could conceivably vary among the samples, affecting the rate of gas escape. This may account for the different morphologies that are observed.

**Figure 4:**
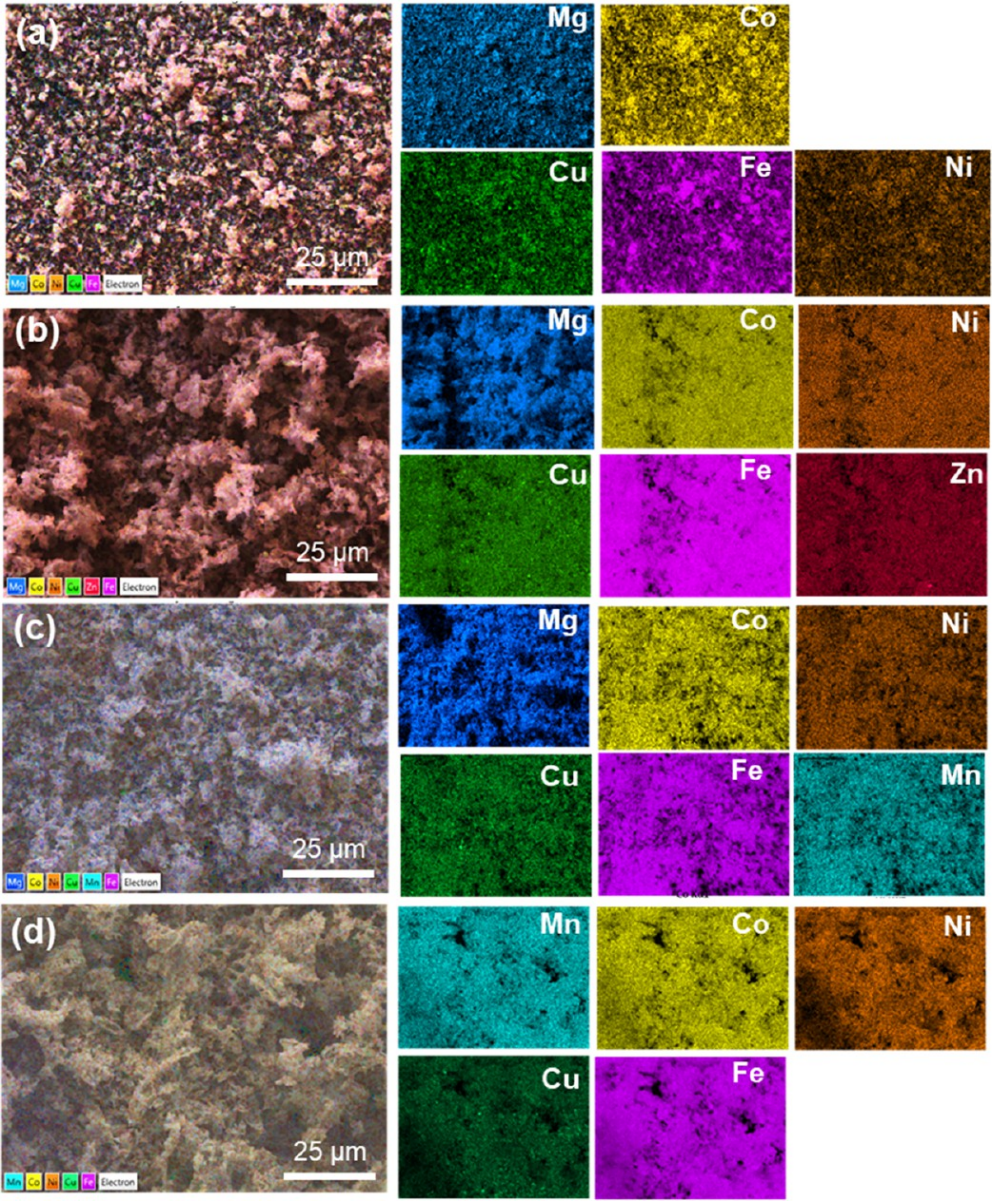
EDS mapping images of (a) HESO-1, (b) HESO-2, (c) HESO-3, and (d) HESO-4.

The elemental distribution of HESOs was characterized by EDS, as shown in Figure 4. The EDS mapping images show uniform element distribution with minor compositional variations.

**Figure 5:**
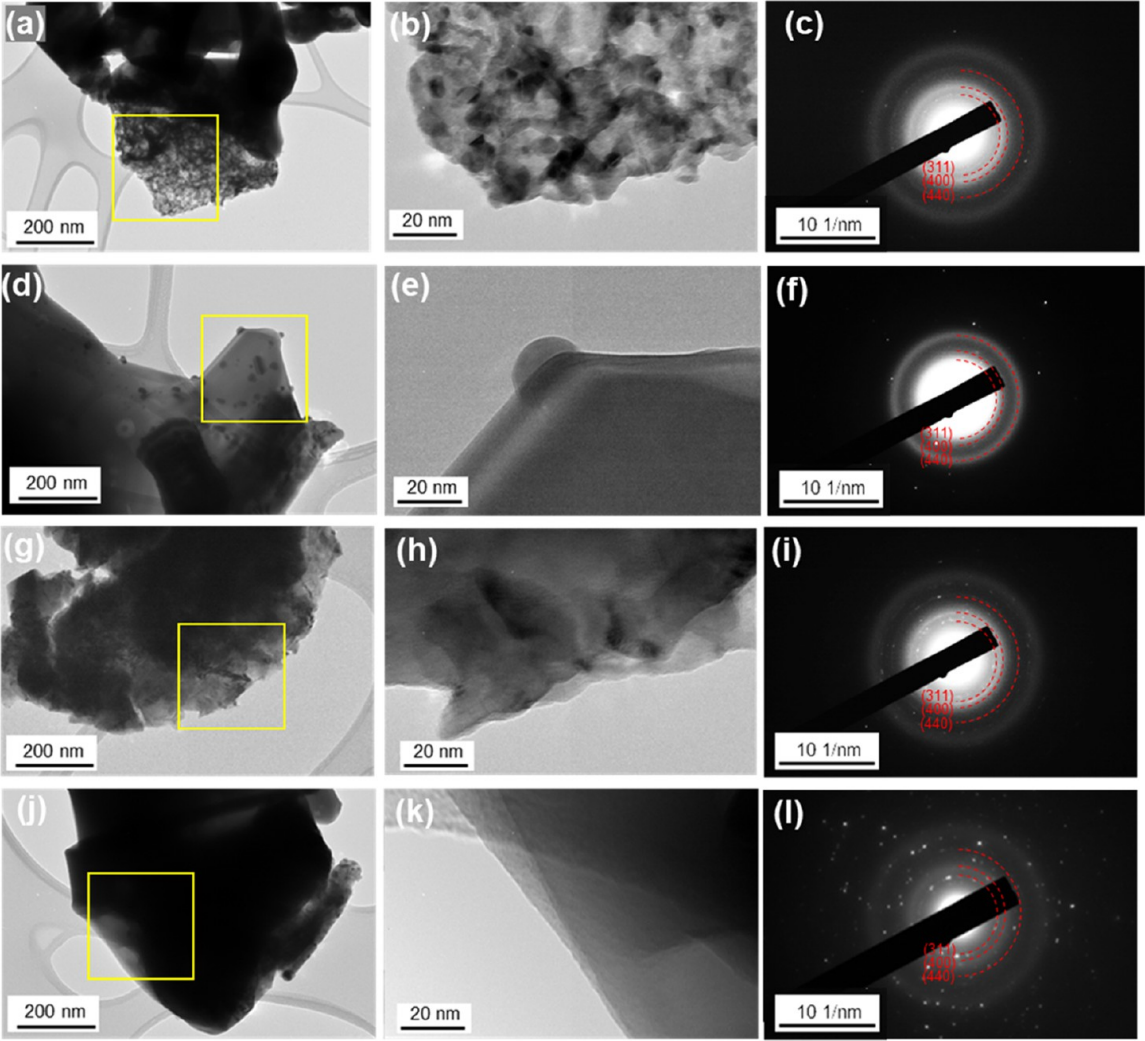
TEM and SAED images of (a–c) HESO-1, (d–f) HESO-2, (g–i) HESO-3, and (j–l) HESO-4. Areas used for SAED are delineated on the TEM images by yellow boxes.

TEM characterization of HESOs is shown in Figure 5. HESO-1 (Figure 5a,b) has a rough surface with particles sintered together, while HESO-2 (Figure 5d,e), HESO-3 (Figure 5g,h), and HESO-4 (Figure 5j,k) are more crystalline and have smoother surfaces. The selected area electron diffraction (SAED) images (Figure 5c,f,i,l) confirmed the crystallinity of the HESOs. The (311), (400), and (440) planes that correspond to the spinel structure are indicated (ICSD code: 184064).

### Electrochemistry

**Figure 6:**
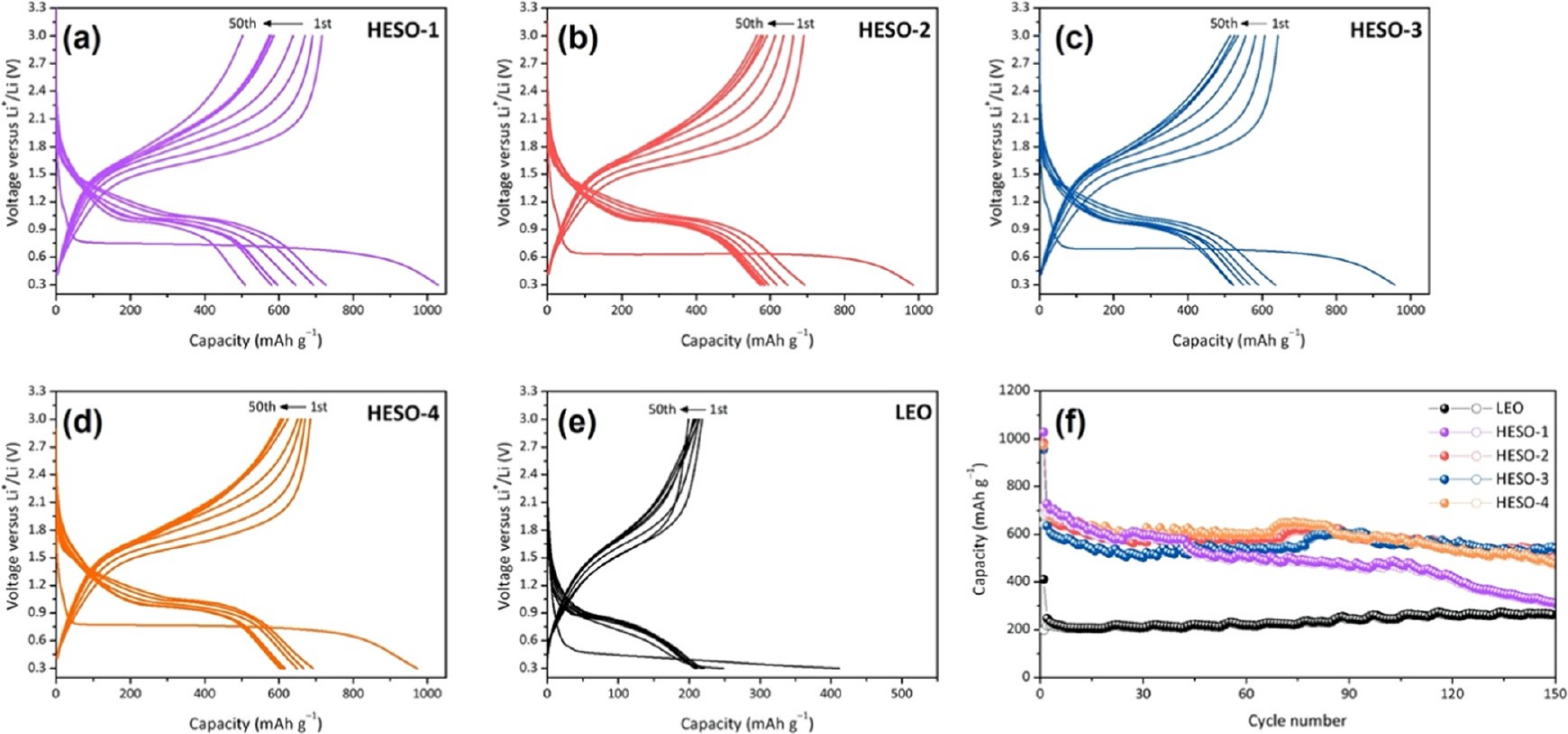
Galvanostatic charge–discharge curves for lithium half-cells containing (a) HESO-1, (b) HESO-2, (c) HESO-3, (d) HESO-4, and (e) LEO (current density: 200 mA g^–1^, voltage window: 0.3–3.0 V). (f) Galvanostatic cycling results on lithium half-cells containing HESO-1, HESO-2, HESO-3, HESO-4, and LEO (current density: 200 mA g^–1^, voltage window: 0.3–3.0 V).

Lithium anode cells were used to evaluate the electrochemical behavior of the HESO and LEO materials. The electrodes were made with CMC binder, and 1 M LiPF_6_ in EC/DEC electrolyte was used in the cells. Figure 6 shows selected galvanostatic charge–discharge curves at a current density of 200 mA g^–1^ between 0.3 and 3.0 V, as well as extended cycling data, and includes results for LEO. CMC binder accommodates large volume changes in active materials better than poly(vinylidene fluoride) (PVdF), as has been recently demonstrated in sodium half-cells for a sodium titanate anode material.^17^

The first discharges of all of the cells resemble one another but differ from the subsequent ones, consisting of a short sloping portion followed by a long plateau at about 0.75 V. Interestingly, this behavior differs from what has been previously observed in Fe_3_O_4_ or MgFe_2_O_4_ nanomaterials in lithium half-cells.^18,19^

The lithiation of Fe_3_O_4_ can be described as a series of steps as described below where the first discharge shows a multistep voltage profile.

1 Reaction of the first equivalent of lithium where Li^+^ inserts into an interstitial octahedral (16c) site.
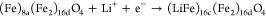
1
2 The second lithium equivalent reacts where Li^+^ ion inserts into the Fe_3_O_4_ lattice, displacing the Li^+^ already situated in 16c sites where the two Li^+^ are redistributed among 8a, 48f, and 8b interstitial tetrahedral sites.
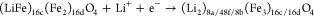
2
3 The third and fourth lithium equivalents react resulting in the formation of Li_2_O·FeO and Fe^0^ metal as described below.
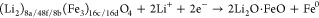
3
4 The reaction of the final four lithium equivalents results in the conversion to iron metal and Li_2_O
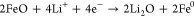
4The overall reaction for lithium and Fe_3_O_4_ is summarized in eq 5.
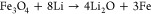
5The lithiation of MgFe_2_O_4_ has also been studied previously.^14,20^ The material structure is a partially inverse spinel where the distribution of cations can vary between octahedral and tetrahedral sites. With the exclusion of Mg^2+^ reduction, the maximum capacity of MgFe_2_O_4_ is 804 mAh g^–1^, corresponding to 6 electron equivalents, as Mg^2+^ is typically not reduced to Mg metal during the discharge process. Upon 2 electron equivalents of lithiation, it has been reported that the MgFe_2_O_4_ electrodes underwent a phase transition from spinel MgFe_2_O_4_ to the rock-salt FeO structure.^18^ Reduction by an additional 4 electron equivalents resulted in Fe^2+^ being fully reduced to metallic Fe^0^. Notably, the theoretical capacity for reduction of Fe_3_O_4_ (eq 5) is 927 mAh g^–1^, higher than that of MgFe_2_O_4_.

The theoretical capacities for the HESOs, assuming that it is possible to reduce all of the metals to the elements, are given in Table S2 and differ only marginally from that of Fe_3_O_4_. The initial discharge capacities obtained for the cells in Figure 6 range from 972 to 1028 mAh g^–1^ for the HESOs and are somewhat higher than expected. The excess capacity upon the first discharge can be explained by irreversible electrolyte decomposition to form a solid electrolyte interphase (SEI). Formation of SEI with resultant capacity in excess of theoretical has been observed in the lithiation of Fe_3_O_4_ where the onset of SEI formation was noted at ∼2 electron equivalents of reduction as verified by a combination of isothermal microcalorimetry or X-ray photoelectron spectroscopy (XPS) with XAS.^21,22^ Capacity upon recharge can give a better estimate of utilization because it is not complicated by the SEI formation phenomenon. Values for the HESOs upon charge range from 68 to 76% of the theoretical capacity. It is not clear, however, that all of the metals can be completely reduced in the HESOs or that they would be completely reoxidized upon recharge. For example, Mn is notoriously difficult to reduce to the elemental form.^23^ This may explain the somewhat lower utilization observed for HESO-3 than for HESO-1, where Mn is substituted for Fe on tetrahedral sites. Replacement of Mg for Mn (HESO-4) does not appear to incur the same penalty, however. An alloying reaction of Mg with Li is theoretically possible, adding capacity to make up for the losses associated with the incomplete reduction of Mn, though it does not appear to be present in previous reports for MgFe_2_O_4_.^18^

The capacities obtained for cells containing LEO were much less than expected, only 18% of the theoretical (assuming all metals are reduced). Here, the plateau on the first cycle occurs at a much lower potential than for the HESO compositions and from what has been observed in previous reports on MgFe_2_O_4_ half-cells,^18,24^ suggesting severe kinetic limitations. MgFe_2_O_4_ can deliver high capacities when used as a conversion anode, but the electrochemical properties are extremely dependent upon particle size, and nanostructuring is required to realize high utilization. This is true of other spinel ferrites as well, e.g., NiFe_2_O_4_.^25^ Nanostructuring can be disadvantageous because the increased surface area of small particles means that more electrolyte is consumed during the formation of the SEI on the first cycle. It may also require more carbon to be added to the composite electrode to ensure good electronic conductivity, reducing practical capacity. The complex morphologies of the materials made by combustion synthesis are apparently not conducive to good electrochemical properties in the case of LEO, although the same does not seem to be true of the HESOs. This suggests that there are benefits stemming from the entropic effects of these materials.

Figure 6 also shows extended cycling data for the HESOs and LEO. There is good capacity retention over 150 cycles for most of the cells with HESO electrodes, with HESO-2, HESO-3, and HESO-4 exhibiting improved capacity retention compared to HESO-1. *Ex situ* XRD patterns of the HESO electrodes after 25 cycles (Figure S6) show that they have become amorphous, which is typical of conversion materials; only peaks attributable to the current collector are observed.

Capacity actually increased slightly for the LEO cells during cycling, indicating a degree of conditioning, although it remained lower than that of all of the HESOs even after 150 cycles and far below the theoretical value.

**Figure 7:**
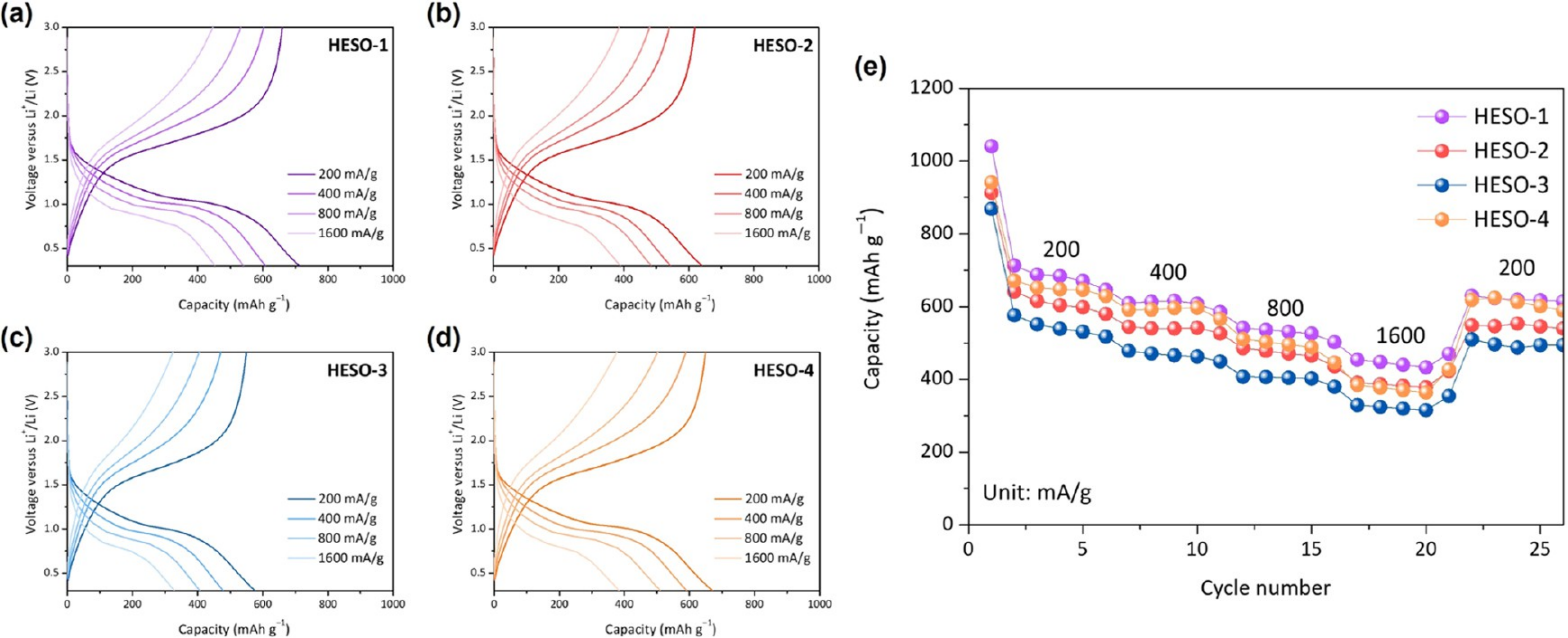
Rate capabilities of cells containing (a) HESO-1, (b) HESO-2, (c) HESO-3 and (d) HESO-4 at the indicated current densities, cycled between 0.3 and 3.0 V. Capacity as a function of cycle number is shown in (e), showing 5 cycles at each current density from 200 to 1600 mA g^–1^, stepped incrementally in doubling, returning to 200 mA g^–1^ for cycles 21–25.

The rate capabilities of HESO cells were evaluated by stepping the current densities from 200 to 1600 mA g^–1^ in increments (Figure 7). In every case, a significant fraction of the capacity (more than half) was maintained even when the current density was increased 8-fold. After five cycles at each current density, the lowest current density of 200 mA g^–1^ was repeated (Figure 7e) and showed good recovery for all of the cells; capacities were comparable in each case to what was obtained upon the second discharges at the same current densities.

**Figure 8:**
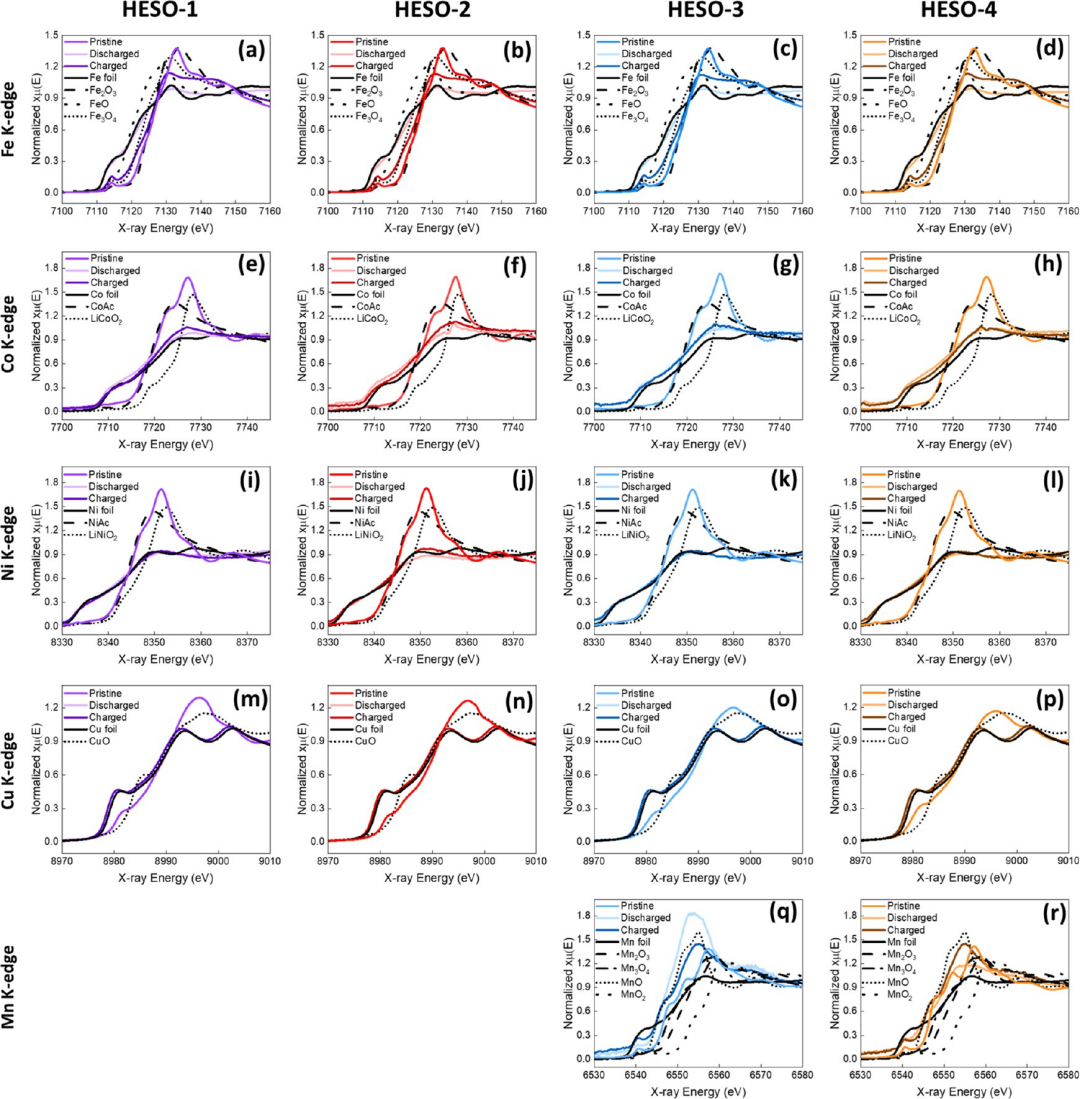
XANES spectra in the pristine, lithiated (discharged), and delithiated (charged) states for (a, e, i, m) HESO-1, (b, f, j, n) HESO-2, (c, g, k, o, q) HESO-3, and (d, h, l, p, r) HESO-4.

Figure 8 shows XANES spectra for HESO electrodes after the first lithiation (discharge) and subsequent delithiation (charge) between 0.3 and 3.0 V in lithium half-cells. Linear combination fitting was used to determine the oxidation states of the Fe, Co, Ni, and Cu metals at each state. An alternative method based on area integration as described in the Experimental Methods was used for the determination of the Mn oxidation states (Figure S3). The average oxidation states for each metal of the HESO materials after electrochemical delithiation or lithiation are shown in Table 5. For the Fe-edge (Figure 8a–d), the energy edge change of the absorption edges of all 4 samples illustrates a similar trend. The energy edges shift to lower energy in the discharged state, indicating an oxidation state around Fe (0) based on comparison with the spectra of Fe foil. In the charged state, the energy edges shift toward higher energy values at the charged state. The edge positions suggest an oxidation state between Fe(III) and Fe(II) when compared to the energy levels of the Fe_2_O_3_ and FeO reference materials. Based on LCF, the pristine electrode had an Fe oxidation state of ∼2.9, which decreased to ∼0.2 after the first discharge and then increased to ∼2.6 after the first charge.

**Table 5:** Average Metal Oxidation States of Electrodes Discharged and Charged between 0.3– and 3.0 V and Stopped at the Indicated State-of-Charge (SOC)

	SOC	Fe	Co	Ni	Cu	Mn
HESO-1	discharged	0.16	0.09	0.0	0.03	
	charged	2.67	0.36	0.06	0.03	
HESO-2	discharged	0.34	0.24	0.0	0.04	
	charged	2.64	0.57	0.08	0.04	
HESO-3	discharged	0.22	0.03	0.0	0.03	1.90
	charged	2.61	0.04	0.0	0.04	2.02
HESO-4	discharged	0.26	0.25	0.0	0.04	2.15
	charged	2.64	0.31	0.0	0.03	2.09

Pre-edge regions of XAS spectra contain information about the oxidation states and coordination of the metal ions that are being probed. In general, the intensities of these features are stronger for noncentrosymmetric coordination (e.g., tetrahedral) than for centrosymmetric (with the caveat that distortions affecting octahedra will also result in greater intensities). The pre-edge regions of XAS spectra at the Fe K-edge of HESO samples are shown in Figure S7. The pre-edges of HESOs were initially compared with Fe-containing standards: Fe foil, FeO, Fe_2_O_3_, and Fe_3_O_4_. In the standard Fe_3_O_4_, only one peak is evident around 7114 eV, whereas the other three spectra appear almost flat. Across these HESO samples, discharged spectra closely resemble that of Fe foil, while charged and pristine spectra exhibit a similar pattern as standard Fe_3_O_4_, featuring a pre-edge at approximately 7114 eV. According to prior references,^26,27^ lower intensity suggests a six-coordinate octahedral (O_h_) site, while higher intensity indicates a four-coordinate tetrahedral (*T*_d_) site. The pristine and charged HESOs exhibit one peak at 7114 eV, which suggests that some Fe is in a *T*_d_ site. This observation is consistent with the EXAFs fitting results where around half of the Fe is in *T*_d_ sites and half of the Fe is in O_h_ sites (Figure S5). The charged HESOs show higher intensity than the pristine at the 7114 eV peak, and the elevated intensity in *T*_d_ coordination indicates a transition from the 1s orbital to the p component within a hybridized d–p orbital.^26^

The Co-edge, Ni-edge, and Cu-edge data (**8e**–**8h**, **8i**–**8l**, and **8m**–**8p**, respectively), for the HESO samples exhibit a similar evolution of oxidation states of Co, Ni, and Cu elements. For the Co energy edge, the oxidation states begin with the oxidation state of ∼2, decreasing to the oxidation state ∼0 in the discharged state in accordance with the edge shifts to lower edge energy. For the charged state, the oxidation state remains similar to the oxidation state obtained in the discharged state. Ni energy edges for all samples shift to lower energy, near that of the Ni foil with the oxidation state of ∼0 in the discharged state. In the subsequent charged state, the Ni energy edge remains ∼0. In the XANES figures for copper, the oxidation states are initially ∼1.8 for the pristine samples. The energy edges shift to lower energy in the discharged state, indicating an oxidation state ∼Cu (0) based on comparison with the spectra of Cu foil. In the charged state, the energy edges minimally shift toward higher energy.

For HESO-3 and HESO-4, the spectra of Mn-edges show some change among the pristine, discharged, and charged states in the XANES region (Figure 8q,r). The Mn XANES for the pristine HESO-3 and HESO-4 indicate that the Mn oxidation states are ∼2.3. In the discharged state, the Mn-edges shift to lower energy, revealing the oxidation state is ∼2 based on comparison with the reference samples for MnO and Mn foil. For the charged state, the oxidation state increased slightly to ∼2.1 based on a comparison of the reference samples of Mn_2_O_3_, Mn_3_O_4_, and MnO. The absorption edge of the pristine HESO-4 exhibits a similar position to HESO-3, but the intensity of the absorption edge of HESO-4 is much higher. For the discharged state, the intensity of the absorption edge is lower than that of HESO-3.

It should be noted that K-edge XAS does not directly probe the valence states (d-electrons) of transition metals but involves excitations from the s to p states. The positions and line shapes of the main edges are affected by changes in environment including variations in coordination, bond lengths, and angles. Thus, different configurations of A-site metals, as in the HESO materials, complicate the interpretation of K-edge data for precise determination of metal oxidation states by comparing to standards. However, trends can still be observed as a function of the oxidation state as evidenced in Figure 2 of ref (28), where obvious groupings of Mn^2+^, Mn^3+^, and Mn^4+^ containing minerals are seen. The changes in the HESO materials are drastic upon delithiation and relithiation, consistent with large swings in the oxidation states and consistent with the electrochemical data, adding confidence to the interpretation of the XANES results.

**Figure 9:**
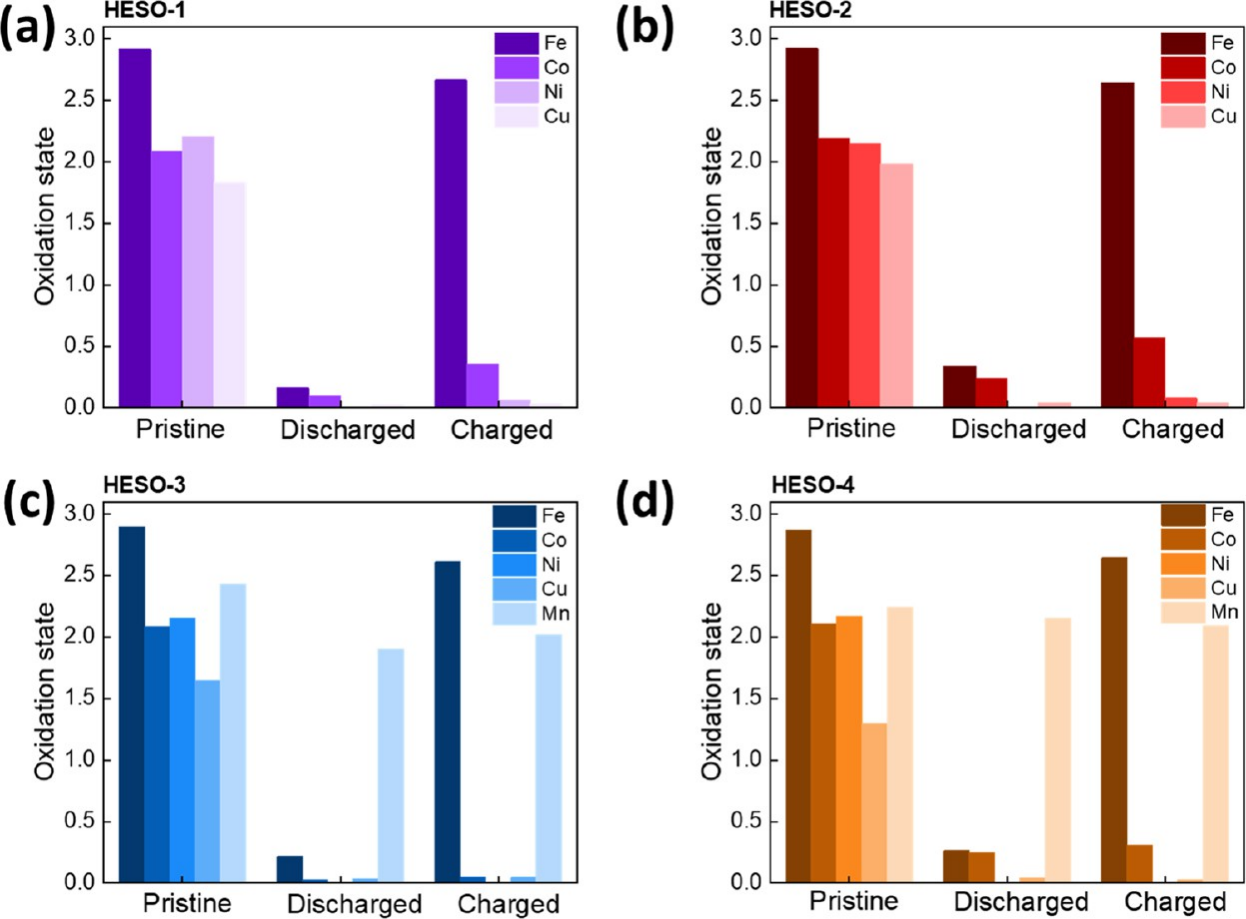
Changes in oxidation states of metals in HESO electrodes, taken from data in Tables 4 and 5; (a) HESO-1, (b) HESO-2, (c) HESO-3, and (d) HESO-4.

Figure 9 summarizes changes in the oxidation state for each of the HESO materials with electrochemical (de)lithiation. The Fe, Co, Ni, and Cu metal centers in the HESO samples are effectively reduced to the metallic state during the lithiation of the HESO materials, while Mn shows less redox activity being reduced only to an oxidation state of ∼2.0. On charge (delithation), the iron centers are oxidized to ∼2.6+, while Co, Ni, and Cu remain reduced and Mn remains near 2.0+. While the reduced metallic components do not contribute to the overall reversible capacity, the presence of reduced metal may provide electrical conductivity through the formation of a conductive *in situ* network. Thus, it is the redox activity of the Fe centers that accounts for virtually all of the capacity reversibly delivered by the HESO systems during cycling.

Notably, the oxidation state of the Fe centers in the HESO samples on charge (delithiation) differs significantly from prior reports of delithiation of ferrite nanomaterials including magnetite, magnesium ferrite (MgFe_2_O_4_), and zinc ferrite (ZnFe_2_O_4_) where the charged (delithiated) Fe centers return only to an oxidation state of Fe^2+^.^18,24,29,30^ In the prior studies of the ferrites, the lithiation mechanism was found to proceed via a [A]_16c_[B_2_]_16d_O_4_ phase with partially occupied 16c sites, where cations rearrange within the spinel framework rather than form pure FeO (or ZnO in the case of ZnFe_2_O_4_) domains. Upon delithiation, the Fe atoms are in a coordination geometry with low inversion symmetry where a significant fraction of the oxidized Fe atoms are in an environment that is distorted from a purely octahedral geometry as would be expected in a rock-salt structure of pure FeO. The spinel ferrites with other transition metal +2 cations (MgFe_2_O_4_, ZnFe_2_O_4_) show phase segregation between the FeO-like phase and either MgO- or ZnO-like domains in the charged state, as determined by local atomic structure analysis of EXAFS data. TEM in conjunction with ab initio calculations was used to probe the in-depth redox mechanism of Fe_3_O_4_, including the occupancies of O^2–^ anions and Li^+^, Fe^2+^, and Fe^3+^ cations at various states of (dis)charge.^30^ The high reversible capacity observed in the HESO materials is related to the ability of the iron to oxidize beyond Fe^2.0^, above that observed for previous ferrites, clearly demonstrating the compositional advantage of these materials.

## Conclusions

HESO ferrites containing 5 or 6 different metals show markedly superior electrochemical properties compared to Fe_3_O_4_ or MgFe_2_O_4_, when used as conversion anodes in lithium half-cells. Capacities in excess of 600 mAh g^–1^ at low rates were obtained after the first cycle and could be maintained for most of the HESOs over 150 cycles. Rate capability was also outstanding, with more than half the low-rate capacity achieved when current density was increased 8-fold. Analysis of pristine, discharged, and charged electrodes using XAS shows that Fe, Co, Ni, and Cu are reduced to the elemental state upon initial discharge (lithiation), while Mn is reduced only slightly. Upon recharge (delithiation), Co, Ni, and Cu remain in the metallic state, while Fe is reoxidized to ∼2.6+. The superior electrochemical properties of the HESOs are attributed to two factors; first, the presence of metallic components in the composite electrodes after the first discharge, which can provide an electronically percolating network, and, second, the ability to oxidize Fe upon charge further in the HESOs than in Fe_3_O_4_ or MgFe_2_O_4_.

## Supporting Information Available

Examples of Mn K-edge XAS fitting using several methods including the integral method; X-ray diffraction pattern of MgFe_2_O_4_ made by combustion synthesis; laboratory XRD patterns of cycled electrodes; EXAFS data and fits; XAS pre-edge data of pristine materials and standards; table of initial capacities and estimated utilization for lithium half-cells containing HESOs and LEO (PDF)
Crystallographic data (HESO-1) (CIF)
Crystallographic data (HESO-2) (CIF)
Crystallographic data (HESO-3) (CIF)
Crystallographic data (HESO-4) (CIF)

## Supplementary Material

cm4c00085_si_001.pdf

cm4c00085_si_002.cif

cm4c00085_si_003.cif

cm4c00085_si_004.cif

cm4c00085_si_005.cif
