# Supplementary material for: High-Entropy Spinel Oxide Ferrites for Battery Applications
Source: Chem Mater. 2024 Apr 30;36(9):4481–94. doi: 10.1021/acs.chemmater.4c00085 (PMC11099913; doi:10.1021/acs.chemmater.4c00085)
Supplement: Supplementary file 1 — cm4c00085_si_001.pdf [file cm4c00085_si_001.pdf]

Supplemental Information

High Entropy Spinel Oxide Ferrites for Battery Applications

Ki-Hun Nam,[a*] Zhongling Wang,[b,c*] Jessica Luo,[b,d] Cynthia Huang,[b,c] Marie F. Millares,[b,c] Alexis Pace,[b] Lei Wang,[b,e] Steven T. King,[b,d] Lu Ma,[f] Steven Ehrlich,[f] Jianming Bai,[f] Esther S. Takeuchi,[b,c,d,e] Amy C. Marschilok,[b,c,d,e] Shan Yan,[b,e] Kenneth J. Takeuchi,[b,c,d,e]** and Marca M. Doeff[a]**

a) Energy Storage and Distributed Resources Division, Lawrence Berkeley National Laboratory, Berkeley CA, 94720 USA
b) Institute of Energy: Sustainability, Environment and Equity, Stony Brook University, Stony Brook, NY, 11794 USA
c) Department of Materials Science and Chemical Engineering, Stony Brook University, Stonybrook, NY, 11794 USA
d) Department of Chemistry, Stony Brook University, Stonybrook, NY, 11794 USA
e) Interdisciplinary Science Department, Brookhaven National Laboratory, Upton, NY, 11973 USA
f) National Synchrotron Light Source II (NSLS II), Brookhaven National Laboratory, Upton, NY, 11973 USA

* co-first authors

**corresponding authors: (K.J.T.) kenneth.takeuchi.1@stonybrook.edu, (M.M.D.) mmdoeff@lbl.gov*corresponding authors: (K.J.T.) kenneth.takeuchi.1@stonybrook.edu, (M.M.D.) mmdoeff@lbl.gov

Method Comparison for determination of Mn Oxidation state for XAS

Several methods were considered for the determination of the Mn oxidation state. The Mn K-edge XAS can be divided into two regions, a small pre-edge around 6542eV and an absorption edge above 6545eV. [1] The main absorption edge arises from the electric dipole-allowed transition from the 1s to 4p level, while the pre-edge originates from two primary transitions. One is the electric quadrupole-allowed and dipole-forbidden1s→3d transition. The probability of the electric quadrupole-allowed transitions is much lower compared with the dipole transition, leading to a much lower intensity in the pre-edge peak unless the coordination environment is non-centrosymmetric due to the mixing between 3d and 4p orbitals.

Three methods are described below. In each case, the methods were applied to the spectra of MnO, Mn_2O_3, MnO_2 and Mn_3O_4 that were used as reference samples. The edge energies were determined and used to establish calibration curves between edge enegy and the oxidation state of Mn. Examples of each method are shown below.

Method 1: Maximum-point of first derivative spectrum Method

The maximum-point method, where the value of edge energy is equal to the highest point energy, as determined by the highest point of the first peak of the first derivative.

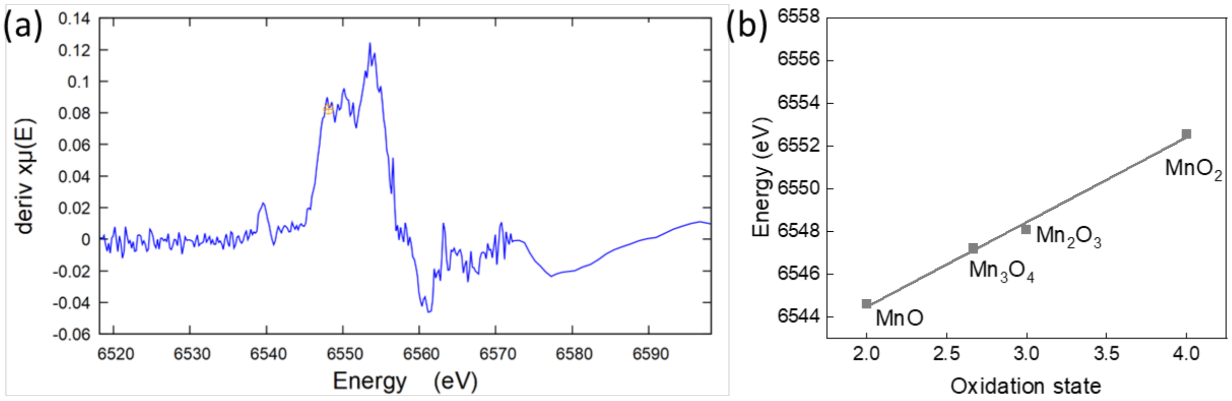

Figure S1. (a) First derivative of the XAS spectrum of Mn_2O_3 with the highest point of the first peak. (b) calibration curve of Mn K-edge energy of Mn standards based on first derivative method.

The linear equation of edge energy (E) obtained from the calibration curve with first derivative method is shown in **equation (1)**, where x is oxidation state and R^2=0.995.

$$\text{Edge energy (E)} = 6536.558 + 3.9624\,x \qquad \textbf{(equation 1)}$$

Method 2: Half-height Method[2]

The value of edge energy is equal to the energy corresponding to a μ-value of 0.5 of the normalized spectra.

Using Mn_2O_3 as an example, the below figure illustrates the edge energy point.

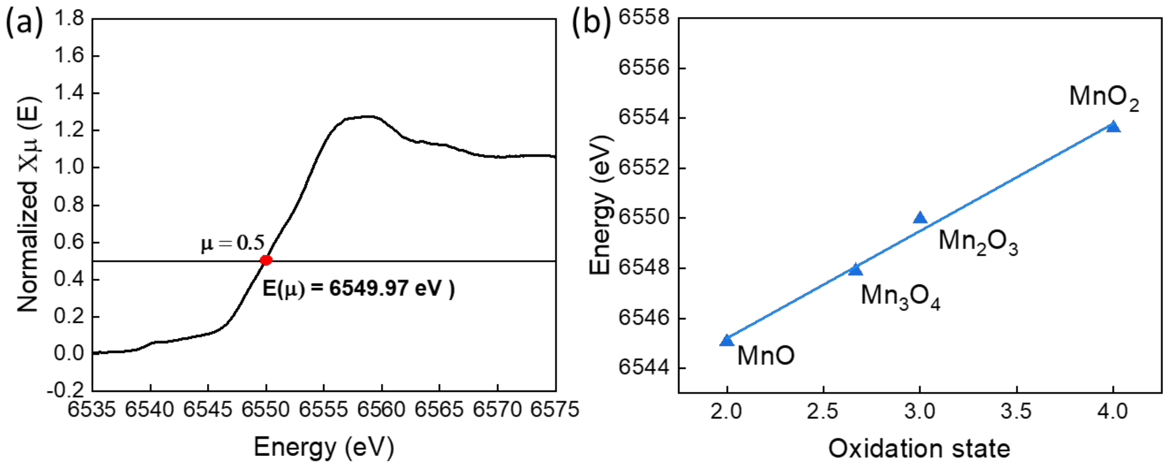

Figure S2. (a) XAS spectrum of Mn_2O_3 with edge energy at half-height. (b) calibration curve of Mn K-edge energy of Mn standards based on half-height method.

The linner equation of edge energy (E) otained from the calibration curve with half height method is shwon in **equation (2)**, where x is oxidation state and R^2=0.992.

Edge energy (E) = 6536.604 + 4.2972 x **(equation 2)**

Method 3: Integral Method[1]

The relationship between position of the absorption threshold at the Mn K-edge and the oxidation state of the metal was established via extraction of the mean value under the spectral area defined by two intensity endpoints, rather than analyses based on single points, based on the equation (3):

$$E = E(m1) + \frac{E(m2) - E(m1)}{m2 - m1} - \frac{S_m}{m2 - m1} \qquad \textbf{(equation 3)}$$

Where m1 and m2 are the upper and lower thresholds of intensity selected for analysis, E(m1) and E(m2) are the corresponding values of energy at these endpoints and S is the area under the spectral curve defined by these four values leading to E = 6551.87 eV.

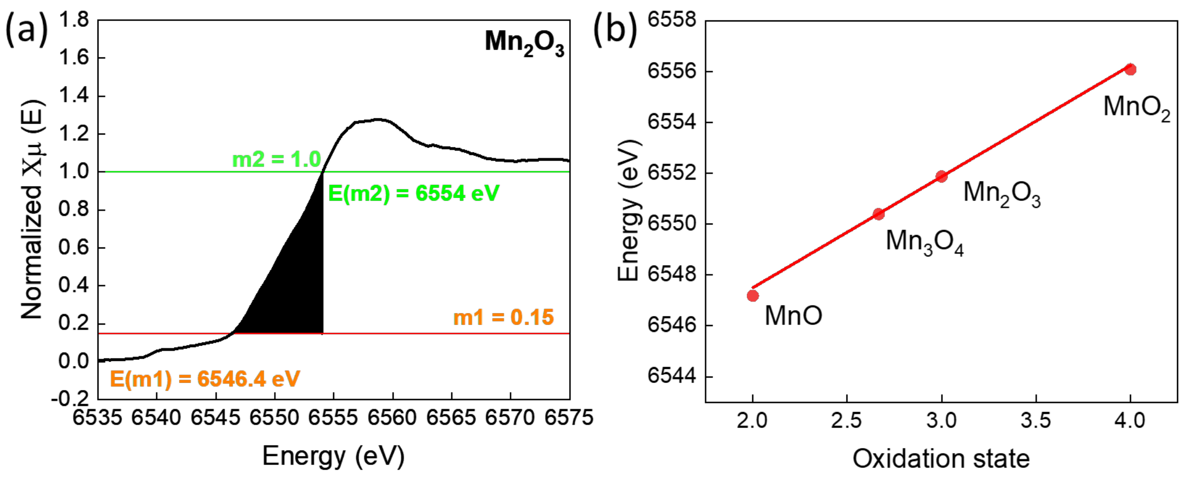

Figure S3. (a) XAS spectrum of Mn_2O_3 with edge energy at intergal area. (b) calibration curve of Mn K-edge energy of Mn standards based on intergral method.

The linear equation of edge energy (E) otained from the calibration curve with integral method is shown in **equation (4)**, where x is oxidation state and R^2=0.999.

$$\text{Edge energy (E)} = 6538.75707 + 4.37012\ x \qquad \textbf{(equation 4)}$$

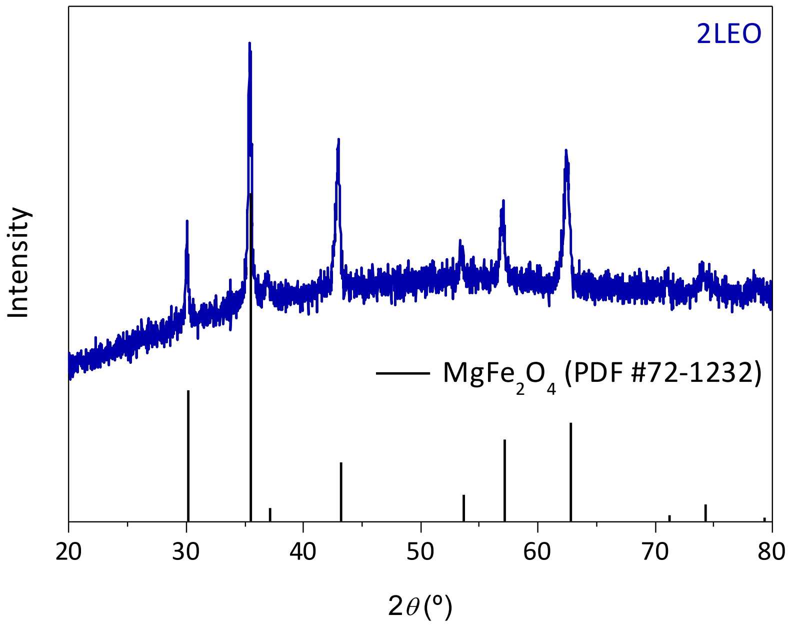

Figure S4. Laboratory XRD pattern of $MgFe_2O_4$ made by combustion synthesis.

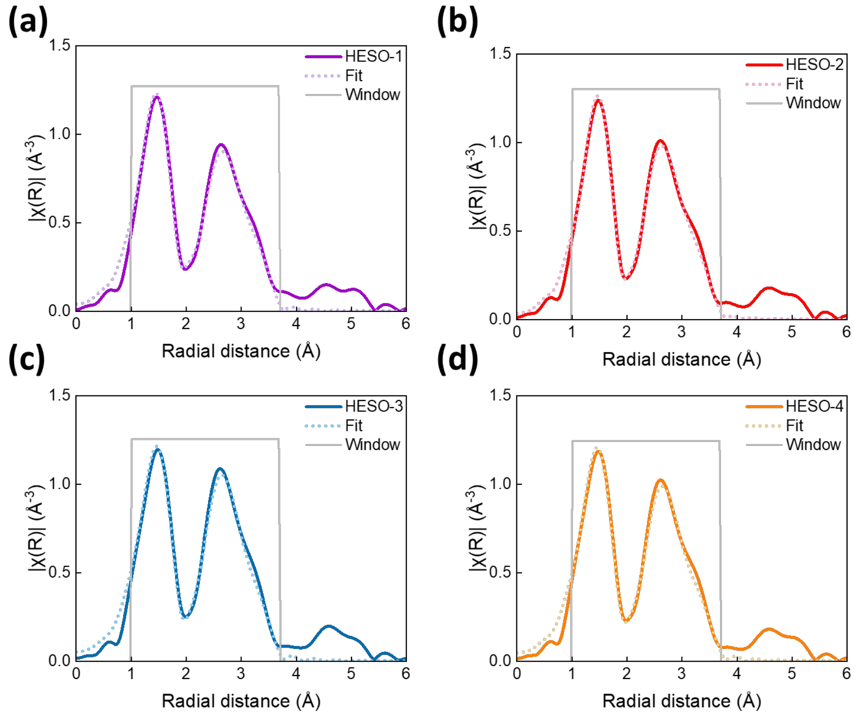

Figure S5. EXAFS fitting results of pristine HESO samples.

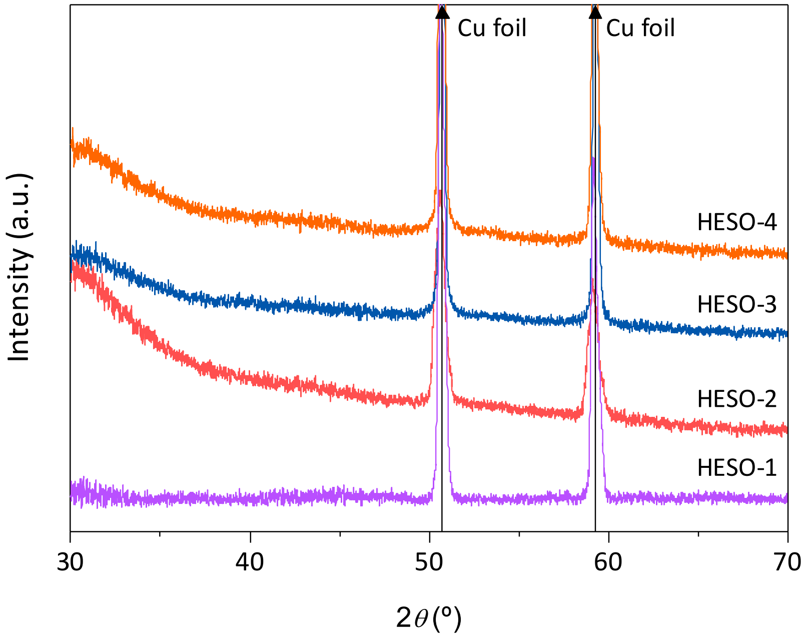

Figure S6. *Ex situ* laboratory XRD patterns of HESO electrodes after 25 cycles.

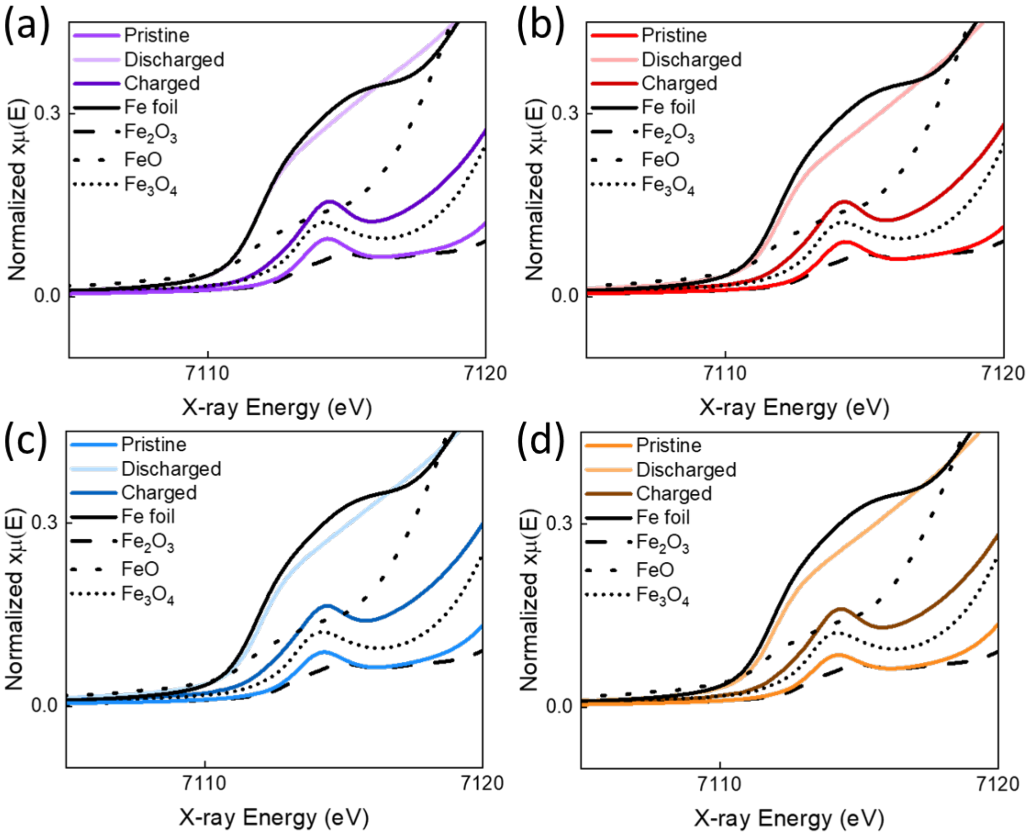

Figure S7. XAS spectra at pre-edge region of HESO samples at Fe K-edge. (a) HESO-1; (b) HESO-2; (c) HESO-3; (d) HESO-4.

Table S1. EXAFS fitting results of pristine HESO samples. [a]

Sample	Core metal position	Phase fraction	Scattering path	S_0^2	N	ΔE_0 (eV)	R (Å)	σ^2	R-factor
**HESO-1**	Tetrahedral	0.5 (1)	Fe-O	**0.875**	**4**	2 (2)	1.88 (2)	0.005 (3)	0.010
			Fe-Fe (Octahedral site)		**12**		3.45 (4)	0.012 (2)	
			Fe-Fe (Tetrahedral site)		**4**		3.53 (8)	0.012 (2)	
	Octahedral	0.5 (1)	Fe-O		**6**	-3 (2)	2.00 (6)	0.005 (3)	
			Fe-Fe (Octahedral site)		**6**		2.97 (1)	0.012 (2)	
			Fe-Fe (Tetrahedral site)		**6**		3.45 (4)	0.012 (2)	
**HESO-2**	Tetrahedral	0.5 (1)	Fe-O	**0.875**	**4**	4 (2)	1.91 (1)	0.006 (5)	0.006
			Fe-Fe (Octahedral site)		**12**		3.48 (2)	0.009 (2)	
			Fe-Fe (Tetrahedral site)		**4**		3.62 (4)	0.009 (2)	
	Octahedral	0. 5 (1)	Fe-O		**6**	-3 (2)	2.01 (1)	0.006 (5)	
			Fe-Fe (Octahedral site)		**6**		2.96 (1)	0.009 (2)	
			Fe-Fe (Tetrahedral site)		**6**		3.48 (2)	0.009 (2)	
**HESO-3**	Tetrahedral	0.5 (0)	Fe-O	**0.875**	**4**	2 (2)	1.88 (0)	0.003 (5)	0.008
			Fe-Fe (Octahedral site)		**12**		3.44 (2)	0.010 (3)	
			Fe-Fe (Tetrahedral site)		**4**		3.54 (6)	0.010 (3)	
	Octahedral	0.5 (0)	Fe-O		**6**	-4 (2)	2.01 (4)	0.003 (5)	
			Fe-Fe (Octahedral site)		**6**		2.97 (0)	0.010 (3)	
			Fe-Fe (Tetrahedral site)		**6**		3.44 (2)	0.010 (3)	
**HESO-4**	Tetrahedral	0.5 (0)	Fe-O	**0.875**	**4**	2 (1)	1.88 (1)	0.003 (0)	0.007
			Fe-Fe (Octahedral site)		**12**		3.45 (1)	0.010 (7)	
			Fe-Fe (Tetrahedral site)		**4**		3.57 (5)	0.010 (7)	
	Octahedral	0.5 (0)	Fe-O		**6**	-4 (1)	2.01 (4)	0.003 (0)	
			Fe-Fe (Octahedral site)		**6**		2.97 (1)	0.010 (7)	
			Fe-Fe (Tetrahedral site)		**6**		3.45 (1)	0.010 (7)	

a) S_0^2: amplitude reduction factor; N: coordination number; ΔE_0: shift in edge energy; R(Å): atomic distance; σ^2: Debye−Waller factor. Values with bold font were kept fixed during the fitting. The amplitude reduction factor is 0.875[3] and the coordination numbers were kept fixed to the values predicted by the symmetry.

Table S2. Initial capacities and estimated utilization for cells containing HESOs and LEO taken from Figure 6.

Material	Nominal Composition	theor. cap. [a] (mAh/g)	1st disch. cap. (mAh/g)	1st ch. cap. (mAh/g)	utilization[b] (%)
HESO-1	$[Mg_{0.2}Co_{0.2}Ni_{0.2}Cu_{0.2}Fe_{0.2}]Fe_2O_4$	941	1028	716	76
HESO-2	$[Mg_{0.2}Co_{0.2}Ni_{0.2}Cu_{0.2}Zn_{0.2}]Fe_2O_4$	933	984	690	74
HESO-3	$[Mg_{0.2}Co_{0.2}Ni_{0.2}Cu_{0.2}Mn_{0.2}]Fe_2O_4$	942	958	642	68
HESO-4	$[Mn_{0.2}Co_{0.2}Ni_{0.2}Cu_{0.2}Fe_{0.2}]Fe_2O_4$	916	972	684	75
LEO	$MgFe_2O_4$	1072	411	198	18

a) Assuming complete reduction of all metals to the elemental state.
b) Utilization defined as charge capacity divided by theoretical capacity.

References

1. Li, H.; Roy, I.; Starczewski, M.; Freeland, J.; Cabana, J., X-ray Absorption Spectroscopy Illustrates the Participation of Oxygen in the Electrochemical Cycling of $Li_4Mn_2O_5$. *J. Phys. Chem. C* **2023,** *127* (17), 7913-7920.
2. Dau, H.; Liebisch, P.; Haumann, M., X-ray absorption spectroscopy to analyze nuclear geometry and electronic structure of biological metal centers—potential and questions examined with special focus on the tetra-nuclear manganese complex of oxygenic photosynthesis. *Anal. and Bioanal. Chem.* **2003,** *376*, 562-583.
3. Bock, D. C.; Pelliccione, C. J.; Zhang, W.; Timoshenko, J.; Knehr, K. W.; West, A. C.; Wang, F.; Li, Y.; Frenkel, A. I.; Takeuchi, E. S.; Takeuchi, K. J.; Marschilok, A. C., Size dependent behavior of Fe_3O_4 crystals during electrochemical (de)lithiation: an in situ X-ray diffraction, ex situ X-ray absorption spectroscopy, transmission electron microscopy and theoretical investigation. *Phys. Chem. Chem. Phys.* **2017,** *19* (31), 20867-20880.
